# Meta-Analysis of the Effect of Mesenchymal Stem Cell Transplantation on Vascular Remodeling after Carotid Balloon Injury in Animal Models

**DOI:** 10.1371/journal.pone.0120082

**Published:** 2015-03-26

**Authors:** Xinxin Ju, Hong Zou, Kejian Liu, Juncang Duan, Shugang Li, Zheng Zhou, Yan Qi, Jin Zhao, Jianming Hu, Lianghai Wang, Wei Jia, Yutao Wei, Yixun Wang, Wenjie Zhang, Lijuan Pang, Feng Li

**Affiliations:** 1 Department of Pathology and Key Laboratory of Xinjiang Endemic and Ethnic Diseases (Ministry of Education), Shihezi University School of Medicine, Shihezi, Xinjiang, China; 2 Department of Cardiology, First Affiliated Hospital to Shihezi University School of Medicine, Shihezi, China; 3 Department of Public Health, Shihezi University School of Medicine, Shihezi, Xinjiang, China; 4 Department of Stomatology, First Affiliated Hospital to Shihezi University School of Medicine, Shihezi, China; 5 Department of Thoracic and Cardiovascular Surgery, Hospital of Xingjian Production and Construction Corps, Wulumuqi, Xinjiang, China; 6 Department of Pathology, First Affiliated Hospital to Shihezi University School of Medicine, Shihezi, Xinjiang, China; University of Miami Miller School of Medicine, UNITED STATES

## Abstract

**Aim:**

A meta-analysis was conducted to assess the efficacy of mesenchymal stem cell (MSC) transplantation in small animal coronary vessels after balloon injury, to provide data for the design of future pre-clinical experiments and human clinical trials.

**Methods:**

The search strategy included the PubMed, EMBASE, Chinese Biomedical Literature (CBM), and China National Knowledge Infrastructure (CKNI) databases. The endpoint was the ratio of vascular neointima/media (I/M). Moreover, neointimal area, re-endothelialization, and proliferating cell nuclear antigen (PCNA) expression were analyzed. Pooled analyses were conducted using random effects models. Heterogeneity and publication bias were also explored. All data were analyzed using RevMan 5.2 and Stata 12.0.

**Results:**

Fifteen studies were reviewed from 238 retrieved animal studies. Compared with controls, MSC transplantation resulted in greater I/M reduction (pooled difference, 0.39; 95% CI, 0.57–0.21; P < 0.0001), greater neointimal area reduction (pooled difference, 0.16; 95% CI, 0.22–0.10; P < 0.0001), decreased PCNA expression (pooled difference, 17.69; 95% CI, 28.94–6.44; P = 0.002), and enhanced re-endothelialization (pooled difference, 3.37; 95% CI, 1.78–4.95; P < 0.0001). The multivariable meta-regression analysis showed that a higher number of transplanted cells (>10^6^; P = 0.017) and later time point of I/M measurement (P = 0.022) were significantly associated with I/M reduction. Subgroup analysis demonstrated a trend for a greater reduction in the ratio of I/M with late MSC transplantation (>1 day), MSCs transplanted through intravenous injection, and atherosclerotic vessels.

**Conclusion:**

The meta-analysis results demonstrate that MSC transplantation might improve injured vascular remodeling. In addition to greater efficacy with a greater number of transplanted MSCs (>10^6^), the long-term effect of MSC transplantation appears to be more significant. The findings of this meta-analysis may help to design future, effective MSC trials.

## Introduction

Percutaneous coronary intervention (PCI) is an effective treatment method for coronary heart disease. However, restenosis after PCI seriously affects the long-term prognosis. Post-angioplasty restenosis is caused largely by smooth muscle cell proliferation and neointimal proliferation, as well as elastic recoil [1]. A number of studies have suggested that inward vascular neointimal remodeling is the main cause of restenosis [2]. There is great potential for stem cells to regenerate damaged tissues in cardiovascular diseases [3]; mesenchymal stem cells (MSCs) could differentiate into functional cell types that are able to repair the diseased or injured tissue in which they are localized [4]. Despite an increase in the number of animal experiments studying the effects of MSCs on the repair of vascular injury, there is variation in not only experimental design but also the results. Animal experiments provide relevant information for clinical practice, while pre-clinical studies have to anticipate if the new therapy is feasible and effective. There are still a number of unknowns regarding MSC therapy in clinical practice, including the effect of MSCs on vascular remodeling after carotid balloon injury, effective number of MSCs, appropriate route, and appropriate timing of cell delivery.

The present study includes a meta-analysis and sub-analysis of data from published animal studies investigating the effect of MSC treatment, to assess the effects of MSC transplantation after carotid balloon injury.

## Materials and Methods

### Eligibility criteria

Two reviewers (XXJ and LJP) independently judged the eligibility of the studies. Eligible studies were randomized controlled trials (RCTs) of carotid balloon injury animal models. Additional requirements included MSC transplantation as the only intervention in the experimental group(s) and comparison with a placebo group. The studies were also required to investigate parameters for vascular remodeling (ratio of vascular neointima/media [I/M], neointimal area, re-endothelialization, and positive expression of proliferating cell nuclear antigen [PCNA]) as final outcomes. Reviews, comments, and editorials were excluded.

### Search strategy

We searched the electronic databases PubMed, EMBASE, Chinese Biomedical Literature (CBM), and China National Knowledge Infrastructure (CKNI) (last search was updated on 5 April 2014) using the following key words and search terms: (mesenchymal stem cell OR mesenchymal stromal cell OR bone marrow stromal cells OR bone marrow mesenchymal stem cells OR mesenchymal progenitor cell) AND (endothelium OR vessel OR vascular) AND (carotid balloon injury OR balloon OR angioplasty).

### Data extraction

Two reviewers (XXJ and LJP) independently screened full-text articles. The following information was extracted from the complete manuscripts of each qualified study: basal characteristics, I/M, neointimal area, re-endothelialization, positive expression of PCNA, time point of I/M measurement, and timing of cell therapy after injury. If necessary, data were estimated from figures of the included studies [5]. The I/M data were all expressed as a fraction of 1 (i.e., data presented as percentage values were converted to a fraction).

### Data analysis

The mean I/M ratios were different between the experimental and control groups. The heterogeneity tests resulted in significant heterogeneity (P < 0.1); therefore, a random-effect model was applied [6]. Multivariable meta-regression analysis was performed to find the source of the significant heterogeneity. Continuous variables were estimated as weighted mean differences with 95% confidence intervals (CIs) between the MSC-treated animals and control animals. In the case of multiple experimental groups compared with one control group within one study, the number of animals in the control group was divided equally by the number of experimental groups [5,7]. P-values were reported by statistical hypothesis testing at the two-sided 0.05 level.

From a clinical viewpoint, multivariate meta-analyses were performed for animal model (rabbits or mice), diet (cholesterol or normal), number of MSCs injected (≤10^6^ or >10^6^), the route of cell delivery (intravenous or carotid artery local transplantation), timing of cell therapy after injury (<1 day or ≥1 day), and time point of I/M measurement after MSC therapy (<4 weeks or ≥4 weeks) to find the factors associated with a reduction in I/M. For further insight, subgroup analyses were performed with the following factors: diet, number of cells injected (≤10^5^, 10^5^–10^6^, 10^6^–10^7^, or >10^7^), the route of cell delivery, timing of cell therapy after injury, and time point of I/M measurement after MSC therapy.

The mean neointimal area, re-endothelialization, and positive expression of PCNA values were also different between the experimental and control groups. The heterogeneity tests resulted in significant heterogeneity (P < 0.1); therefore, a random-effect model was applied. The degree of re-endothelialization was assessed using 3 methods: the expression of CD31, vascular endothelial growth factor (VEGF), and ratio of the re-endothelialized area (defined as the area not stained with Evans blue/total injured surface area).

Publication bias was explored by funnel plot. All analyses were performed with Review Manager Version 5.2 (The Nordic Cochrane Centre, The Cochrane Collaboration, 2012) and Stata 12.0.

## Results

### Study characteristics

**Fig 1:**
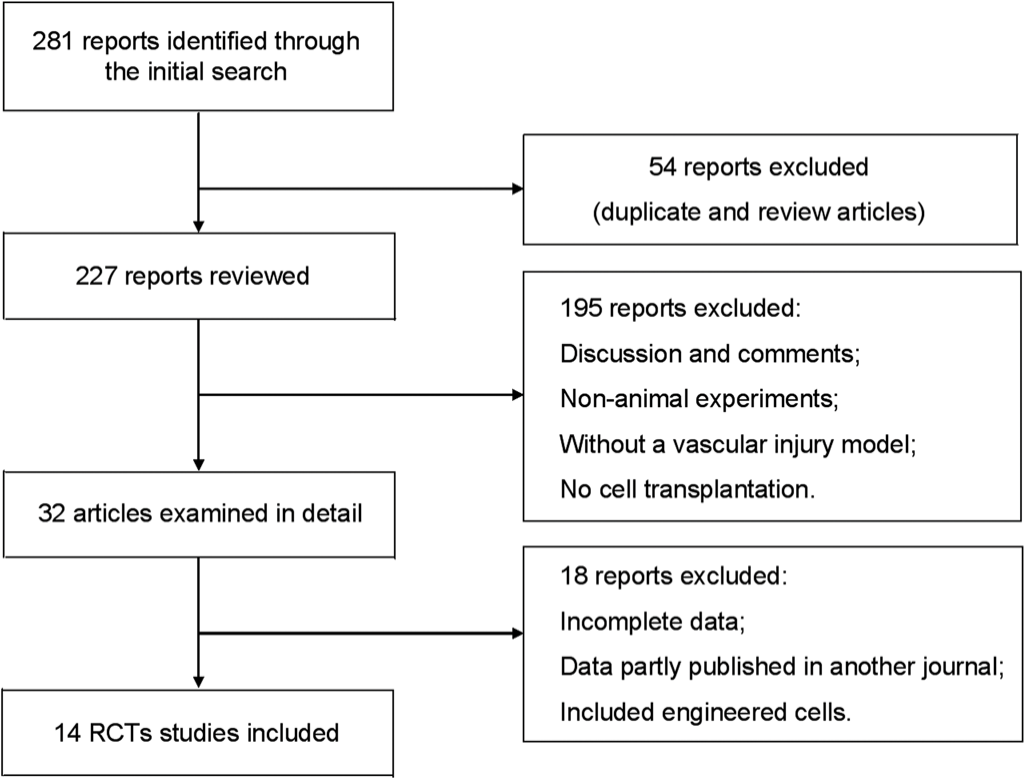
Flowchart of studies included in the meta-analysis of animal studies investigating mesenchymal stem cell therapy. Abbreviations: RCTs = randomized controlled trials.

The search strategy identified 281 articles (Fig. 1). Of the 238 animal studies, 14 were eligible for review (Table 1). Mice (7 studies) and rabbits (7 studies) were used as animal models. The studies investigated the effect of MSC on injured atherosclerotic vessels (6 studies) or injured normal vessels (8 studies). The atherosclerotic vessel models resulted primarily from animals fed a cholesterol diet, while the normal vessel models resulted primarily from animals fed a normal diet. MSCs were injected either intravenously (n = 4) or near the injured carotid artery (n = 10). The MSC doses varied, as summarized by 2 categories: low dose (≤10^6^) in 4 studies and high dose (>10^6^) in 9 studies. Timing between vessel injury and cell transplantation was <1 day (12 studies) or ≥1 day (2 studies). After cell transplantation, the I/M level was measured at <4 weeks (4 studies) or ≥4 weeks (12 studies).

**Table 1:** Characteristics of the animal studies included in the meta-analysis.

First author (year)	n	Type of animal	Diet	Number of MSCs	Route of delivery	Time of MSC therapy after injury	Time point of I/M measurement
Chen (2007)[8]	6	Mice	Normal	2×10^6^	Carotid artery	<1 day	28 days
Guo (2010)[9]	10	Rabbits	Cholesterol	2×10^7^	Intravenous	<1 day	28 days
Hou (2009)[10]	8	Mice	Cholesterol	1×10^6^	Carotid artery	<1 day	60 days
Liu (2009)[11]	5	Rabbits	Normal	1×10^5^	Carotid artery	<1 day	28 days
Liu 1 (2013)[12]	10	Rabbits	Cholesterol	2×10^7^	Carotid artery	<1 day	28 days
Liu 2 (2013)[13]	12	Mice	Normal	5×10^7^	Intravenous	<1 day	28 days
Liu (2010)[14]	8	Rabbits	Cholesterol	2×10^7^	Carotid artery	<1 day	28 days
Long (2012)[15]	6	Rabbits	Cholesterol	1.5×10^7^	Intravenous	≥1 day	28 days
Long (2014)[16]	6	Rabbits	Normal	1×10^8^	Intravenous	≥1 day	14 days or 28 days
Miao (2010)[17]	6	Mice	Normal	5×10^2^	Carotid artery	<1 day	14 days or 28 days
O’Shea (2010)[18]	6	Rabbits	Normal	1×10^5^	Carotid artery	<1 day	14 days
Shu (2012)[19]	8	Mice	Normal	5×10^6^	Carotid artery	<1 day	28 days
Xu (2010)[20]	6	Mice	Normal	2×10^6^	Intravenous	<1 day	28 days
Liao (2012)[21]	8	Mice	Cholesterol	Not reported	Carotid artery	<1 day	42 days

### Meta-analyses

**Fig 2:**
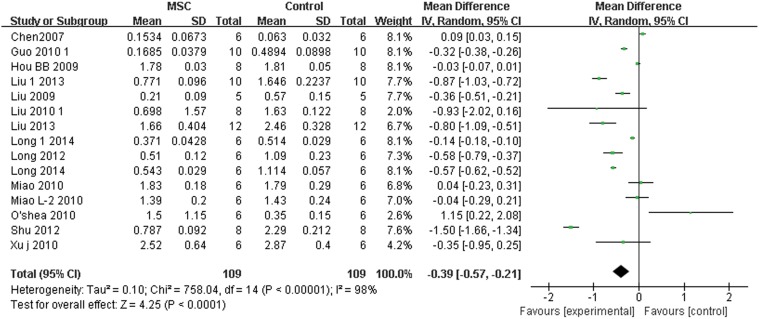
Forest plot showing the impact of mesenchymal stem cell (MSC) therapy on neointima/media, compared with controls. Abbreviations: SD = standard deviation, IV = independent variable, 95% CI = 95% confidence interval.

The I/M data are presented as continuous variables, using mean and standard deviation. Pooled analysis showed that the I/M level was 0.39 lower in the experimental group compared with the control group after cell therapy (95% CI, 0.57–0.21; Z = 4.25; P < 0.0001) with significant heterogeneity (P < 0.001; I Square, 98%; Fig. 2).

Multivariable meta-regression analysis showed that the number of MSCs transplanted (P = 0.017) and time point of I/M measurement after cell therapy (P = 0.022) were significantly associated with a reduction in I/M.

**Fig 3:**
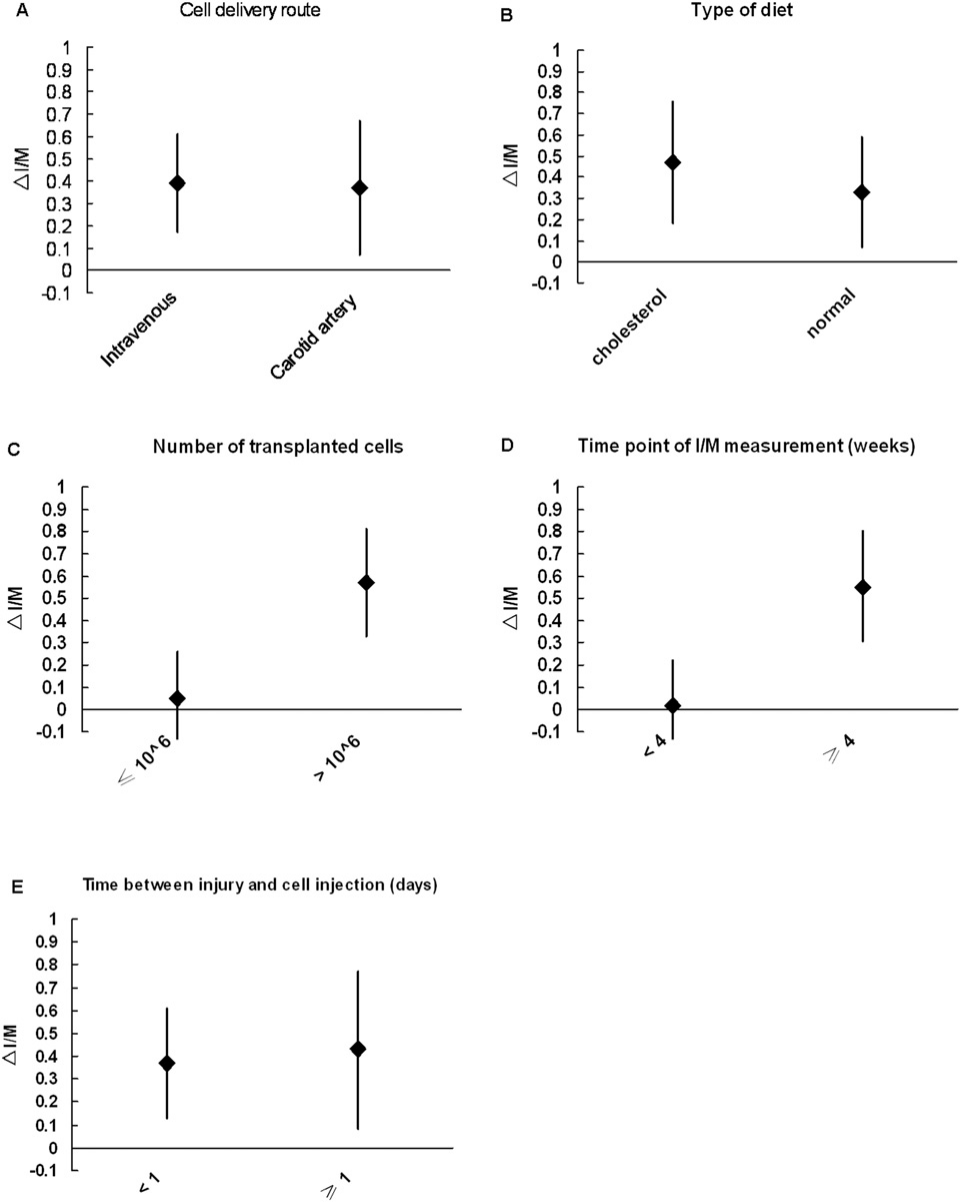
Sensitivity analysis showed a trend towards greater reduction in neointima/media (I/M) with mesenchymal stem cell (MSC) engraftment, compared with controls, with (A) intravenous cell transplantation route (P = 0.139); (B) cholesterol diet (P = 0.629); (C) high cell numbers (>10^6^; P = 0.017); (D) I/M measurement later after MSC engraftment (≥4 weeks; P = 0.022); and (E) later cell engraftment (≥1 day; P = 0.437).

Fig. 3 shows the results of the subgroup analyses. In studies with low MSC doses (≤10^6^), the I/M (95% CI, -0.15–0.26) was 0.05 lower in the MSC group versus the control group. In studies with high MSC doses (>10^6^), the I/M was significantly lower (mean difference, 0.57; 95% CI, 0.33–0.81; P < 0.001). The sub-analyses also demonstrated that the I/M measured at 14 days (<28 days) was 0.02 (95% CI, -0.18–0.22) lower in the MSC group compared with the control group, while the I/M measured at ≥28 days was significantly lower, by 0.55 (95% CI, 0.31–0.80; P < 0.001). The studies that used an intravenous injection route showed a 0.39 lower I/M in the MSC group compared with the control group (95% CI, 0.17–0.61; P < 0.001), while the arterial injection route resulted in a 0.37 (95% CI, 0.07–0.67; P < 0.001) lower I/M. After carotid balloon injury, atherosclerotic vessels had significantly lower I/M than normal vessels in the MSC group compared with the control group (mean difference 0.47 and 0.33, respectively; 95% CI, 0.18–0.76 and 0.07-–0.59, respectively; P < 0.001). Early stage (<1 day) MSC therapy resulted in significantly lower I/M (mean difference, 0.37; 95% CI, 0.13–0.61; P < 0.001) in the MSC group versus the control group. However, late stage (≥1 day) MSC transplantation resulted in significantly lower I/M than early stage (mean difference, 0.43; 95% CI, 0.08–0.77; P < 0.001).

Studies using rabbits resulted in significantly lower I/M in the MSC group versus the control group (mean difference, 0.42; 95% CI, 0.22–0.62; P < 0.001), and studies with mice showed a smaller difference in I/M (mean difference, 0.37; 95% CI, 0.04–0.70; P < 0.001).

**Fig 4:**
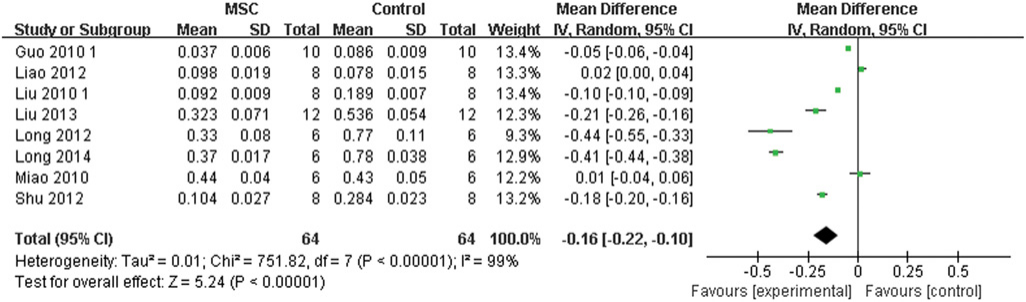
Forest plot showing the impact of mesenchymal stem cell (MSC) therapy on neointimal area, compared with controls. Abbreviations: SD = standard deviation, IV = independent variable, 95% CI = 95% confidence interval.

**Fig 5:**
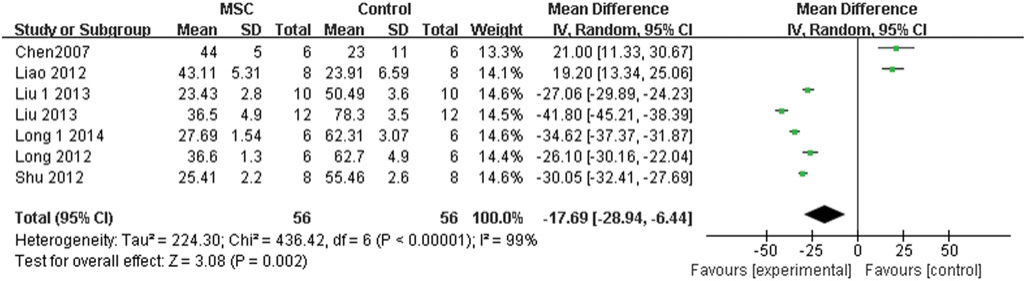
Forest plot showing the impact of mesenchymal stem cell (MSC) therapy on the positive expression of proliferating cell nuclear antigen (PCNA), compared with controls. Abbreviations: SD = standard deviation, IV = independent variable, 95% CI = 95% confidence interval.

The mean neointimal area and positive expression of PCNA are presented as continuous variables, using mean and standard deviation. Pooled analysis showed that the neointimal area was 0.16 lower in the MSC group compared with the control group (95% CI, 0.22–0.10; Z = 5.24; P < 0.0001; Fig. 4). The positive expression of PCNA was 17.69 lower in the MSC group compared with the control group (95% CI, 28.94–6.44; Z = 3.08; P = 0.002; Fig. 5).

**Fig 6:**
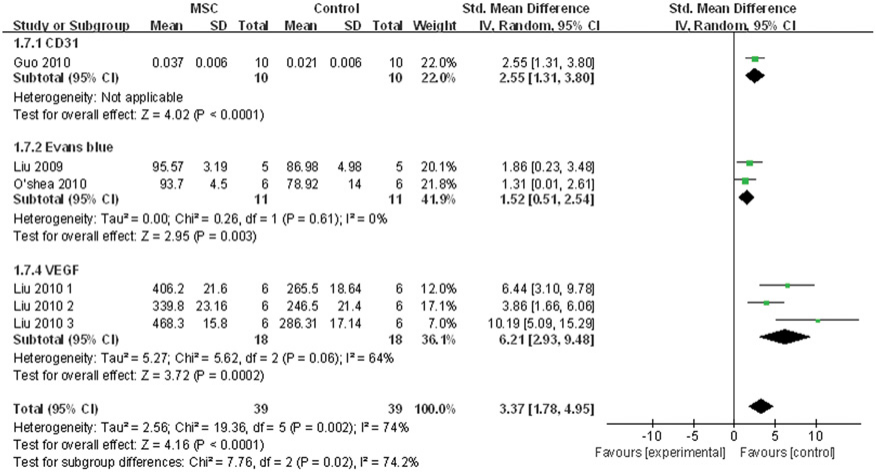
Forest plot showing the impact of mesenchymal stem cell (MSC) therapy on re-endothelialization, compared with controls. CD31, Evans blue, and vascular endothelial growth factor (VEGF) were used as the indices reflecting the degree of re-endothelialization. Abbreviations: SD = standard deviation, IV = independent variable, 95% CI = 95% confidence interval, CD31 = the expression ratio of CD31, VEGF = vascular endothelial growth factor, Evans blue = the ratio of re-endothelialized area (defined as the area not stained with Evans blue/total injured surface area).

Fig. 6 shows the degree of re-endothelialization with each of the 3 assessment methods. Pooled analysis showed that MSC engraftment could enhance vascular re-endothelialization after carotid balloon injury (pooled difference, 3.37; 95% CI, 1.78–4.95; Z = 4.16; P < 0.0001).

### Sensitivity analysis

**Fig 7:**
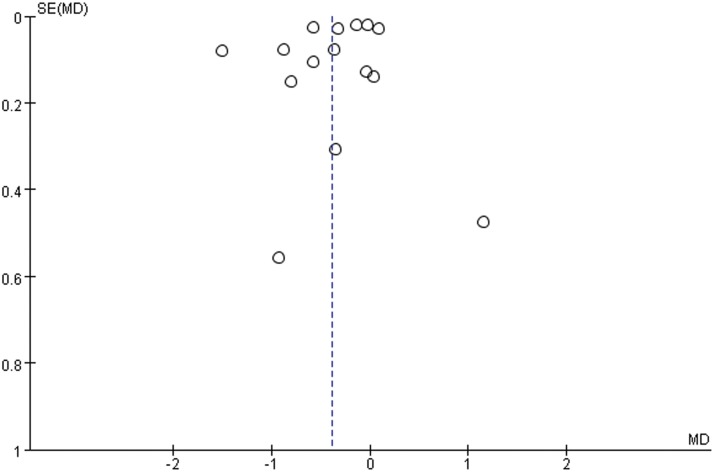
Funnel plot for neointima/media. Blue-dotted line shows the overall estimated mean difference. No obvious evidence for publication bias was found. Abbreviations: MD = mean difference, SE = standard error.

The number of cells transplanted (P = 0.017) and time point of I/M measurement after MSC therapy (P = 0.022) were significant factors for I/M reduction. A trend was observed (Fig. 3) for greater improvement with MSC engraftment with a higher number of cells transplanted (>10^6^) and later engraftment (≥1 day). Local MSC engraftment, around the carotid artery, showed less effect than intravenous engraftment. Animals fed with cholesterol had greater reductions in I/M than animals fed a normal diet. During the follow-up, the late effect of MSC transplantation appeared to be more significant. No trend in I/M differences was observed based on animal type (P = 0.914). The funnel plot for I/M suggests a lack of publication bias, as values were evenly distributed around the overall estimate (Fig. 7).

## Discussion

The present meta-analysis revealed that MSC engraftment reduces vascular remodeling after coronary vessel balloon injury, evidenced by lower/smaller I/M, neointimal area, and positive expression of PCNA in addition to enhanced re-endothelialization after MSC transplantation. In addition, the meta-analysis revealed that that the number of transplanted MSCs and time point of I/M measurement were significant factors for vascular remodeling. Furthermore, the subgroup analysis indicated that late therapy (>1 day), the atherosclerotic vascular model, and intravenously injected MSCs were beneficial for I/M reduction. These results were not affected by the type of animal studied.

MSCs are multipotent cells derived from bone marrow that have the ability to differentiate into cells of mesenchymal and nonmesenchymal origin [22], such as osteocytes [23], chondrocytic, adipocytes [24,25] and myocytes [26]. Moreover, MSCs appear to function through paracrine mechanisms that exert immunosuppressive, anti-inflammatory, anti-apoptotic and other organ-protective and repair-stimulating actions [22,27,28]. As a candidate therapy, MSCs have many advantages [22,29], including easily isolated and amplified from the bone marrow [30] and immunologically tolerated as an allogeneic transplant [29,31].

A number of studies have indicated that MSCs are candidates for cell therapy in coronary heart disease, including myocardial infarction (MI) [32] and ischemic heart disease [33]. With regards to MI, MSCs might improve angiogenesis after MI, reduce MI size, and improve heart function, as demonstrated through animal studies [34] and clinical use [35–37]. As a result, studies have also investigated the effect of MSCs on restenosis after PCI; however, the results have been inconclusive, and MSC use has not been applied clinically.

The mechanisms of vascular restenosis after PCI have remained unclear, while it is known that restenosis after angioplasty is caused largely by smooth muscle cell proliferation and neointimal proliferation, as well as elastic recoil [1]. Inward remodeling might also contribute to restenosis [2]. Some studies have shown that MSCs can improve vascular remodeling and prevent neointimal formation [9–17,19,20], while other studies found that MSCs increase neointimal formation and aggravate vascular remodeling [8,18,21].

The present meta-analysis, which analyzed the I/M, neointimal area, positive expression of PCNA, and degree of re-endothelialization as parameters indicating vascular remodeling after carotid balloon injury, showed that MSC transplantation could improve vascular remodeling and prevent neointimal formation. Furthermore, the I/M results suggested that vascular remodeling is significantly influenced by the number of MSCs transplanted, with a higher number of cells (>10^6^) resulting in a greater reduction in I/M. This result supports those of other meta-analyses regarding the effect of MSC transplantation on other clinical diseases [5,7]. Collectively, these findings indicate that future studies of MSC therapy for restenosis should focus on transplantation of a higher number of MSCs (>10^6^).

I/M reduction was also influenced by the time point of measurement; a greater reduction in I/M was measured at later times (≥4 weeks) after MSC injection. It may be likely that the reduction in intimal remodeling with MSC therapy is only evident over time. However, the results of MSC therapy for ischemic heart disease in pre-clinical studies do not agree with the present findings [5]. The follow-up duration in these studies may not have been long enough; the majority of the studies followed the patients up for no more than 4 weeks, while only one study measured I/M at 5 weeks [10]. Therefore, the long-term effect of MSC therapy on intimal remodeling was previously unknown. Future pre-clinical studies should study the effectiveness of MSC treatment over a longer period.

The subgroup analysis indicated that a greater benefit in intimal remodeling is realized with MSC injections administered ≥1 day after vascular injury. In the acute setting (<24 h), cellular retention and survival are likely influenced by the local hostile microenvironment. Furthermore, MSC therapy tended to be more effective with injured atherosclerotic vessels than with injured normal vessels. Meanwhile, the effectiveness of MSC transplantation was similar between intravenous and carotid artery delivery routes as well as between animal types. Further research is required to determine if there is a best administration route, while the efficacy of MSC may be the same regardless of species (animal or human).

Meta-analyses of animal studies are not common, yet they are recommended in many settings and can often guide research, even clinical endeavors. Performing meta-analyses of animal studies may be a useful way to evaluate the effects of therapies for designing future animal experiment and pre-clinical trials. Our meta-analysis is the first systematic review and meta-analysis of animal models to evaluate the effects of MSC therapy in injured vessels, demonstrating that MSC therapy can lead to improvements in vascular remolding. Moreover, we analyzed the factors influencing vascular remodeling after balloon injury, which could form the basis for future large animal experiments and even human clinical trials. To date, trials for MSC therapy have been performed primarily in small animals. In our opinion, more animal experiments of different species, especially nonhuman primate trials, should be performed in order to assess the efficacy and safety of MSC therapy before human clinical trials.

### Limitations

A limitation of the present study was the presence of obvious heterogeneity. Furthermore, the analysis was based on study outcomes; we did not have access to individual data. However, the risk of erroneous estimates was minimized through the use of random-effect analysis. The observed outcomes were influenced by many factors, such as the diversity in animal type, vascular conditions, cell delivery method, number of cells, time of injection after balloon injury, and follow-up after cell therapy. We performed multivariate analysis, as an exploratory tool, that showed the number of cells and follow-up duration after cell therapy could influence the meta-analysis results. Despite these limitations, our results are highly relevant for future clinical trials.

## References

[1] Grech ED. ABC of interventional cardiology: percutaneous coronary intervention. I: history and development. BMJ. 2003;326: 1080–1082. 12750213 10.1136/bmj.326.7398.1080 PMC1125993

[2] Pasterkamp G, de Kleijn DP, Borst C. Arterial remodeling in atherosclerosis, restenosis and after alteration of blood flow: potential mechanisms and clinical implications. Cardiovasc Res. 2000;45: 843–852. 10728409 10.1016/s0008-6363(99)00377-6

[3] Rajala K, Pekkanen-Mattila M, Aalto-Setala K. Cardiac differentiation of pluripotent stem cells. Stem cells international. 2011;2011: 383709 10.4061/2011/383709 21603143 PMC3096314

[4] Forte A, Galderisi U, Cipollaro M, Cascino A. Mesenchymal stem cells: A good candidate for restenosis therapy? Curr Vasc Pharmacol. 2009;7: 381–393. 19601863 10.2174/157016109788340776

[5] van der Spoel TI, Jansen of Lorkeers SJ, Agostoni P, van Belle E, Gyongyosi M, Sluijter JP, et al Human relevance of pre-clinical studies in stem cell therapy: systematic review and meta-analysis of large animal models of ischaemic heart disease. Cardiovasc Res. 2011; 91: 649–658. 10.1093/cvr/cvr113 21498423

[6] Higgins JP, Green S. Cochrane Handbook for Systematic Reviews of Interventions: Cochrane Book Series. John Wiley & Sons 2008;Ltd 10.

[7] Wang Y, He J, Pei X, Zhao W. Systematic review and meta-analysis of mesenchymal stem/stromal cells therapy for impaired renal function in small animal models. Nephrology (Carlton,). 2013;18: 201–208. 10.1111/nep.12018 23217027

[8] Chen XC, Shan HW, Qu HL, Ge JB, Ge ZP. Bone marrow mesenchymal stem cell transplantation aggravates postangioplasty aortic restenosis in rats. Chinese Journal of Cardiology. 2007;35: 802–806. 18070470

[9] Guo Y, Shi B, Wang ZL, Wang DM, Xu GX. Effects of peripheral blood mesenchyme stem cells transplantation on vascular smooth muscle cell apoptosis after balloon-induced artery injury in rabbits. Chinese Journal of Biomedical Engineering. 2010;29: 288–294.

[10] Hou BB, Wang YQ, Guo JC, Wang M, Zhang P. Effects of lentivirus-mediated angiopoietin-1 gene modified bone marrow mesenchymal stem cells transplantation on carotid atherosclerosis. Journal of Clinical Rehabilitative Tissue Engineering Research. 2009;13: 6309–6313.

[11] Liu P, Zhang L, Liao W, Du W, Gu D, Liu M, et al Allogeneic mesenchymal stem cells confer vascular protection after balloon angioplasty in rabbit carotids. Journal of Thrombosis and Haemostasis. 2009;7: 226.

[12] Liu ZJ, Shi B, Xu GX, Zhao YZ, Shen GY, Wang ZL, et al Effect of Bone Marrow Mesenchymal Stem Cells Transplantation on Vascular Restenosis After Carotid Balloon Injury in Rabbits. Chin J Arterioscler. 2013;21: 22–27.

[13] Liu ZJ, Shi B, Zhao YZ, Shen GY, Chen PK. Effects of CXC receptor 4 gene-modified bone marrow mesenchymal stem cells transplantatiOn on repairment of carotid injure in rats. Chin J Geriatr. 2013;32: 996–1000.

[14] Liu ZJ, Shi B, Xu GX, Zhao RZ, Shen CY, Wang DM, et al Effect of bone marrow mesenchymal stem cells transplantation on expression of NFB and PCNA and vascular steNOSis after carotid artery balloon injury of rabbit. Heart. 2010;96: A24.

[15] Long XP, Zhao RZ, Shi B, Xu GX, Shen CY. Effects of hRAMP1 modified mesenchymal stem cells on restenosis and heart function in rabbit model of carotid angioplasty and myocardial infarction. National Medical Journal of China. 2012;92: 2134–2139. 23158279

[16] Shi B, Long X, Zhao R, Liu Z, Wang D, Xu G, et al Featured Article: Transplantation of mesenchymal stem cells carrying the human receptor activity-modifying protein 1 gene improves cardiac function and inhibits neointimal proliferation in the carotid angioplasty and myocardial infarction rabbit model. Experimental Biology and Medicine. 2014;239: 356–365. 10.1177/1535370213517619 24477823

[17] Jing T, Miao L, He G, Wang H, Liu J, Song Z, et al Conditioanl expression OF the type 2 angiotensin II receptor inhibits neointima formation after carotid injury in rats. Circulation. 2010;122: e288–e289.

[18] O'Shea CA, Hynes SO, Shaw G, Coen BA, Hynes AC, McMahon J, et al Bolus delivery of mesenchymal stem cells to injured vasculature in the rabbit carotid artery produces a dysfunctional endothelium. Tissue Eng Part A. 2010;16: 1657–1665. 10.1089/ten.TEA.2009.0468 20001215

[19] Bo S, Fang F. Effect of bone marrow mesenchymal stem cells transplantation on intimal hyperplasia and the expression of inflammatory cytokines in rats after angioplasty. China Journal of Gerontology. 2012;32: 2989–2992.

[20] Xu J, Gao PJ, Wu DJ, Chen QZ. Endothelial-like cells derived from mesenchymal stem cells attenuate neointimal hyperplasia after vascular injury. Journal of Hypertension. 2010;28: e348–e349.

[21] Liao J, Chen X, Li Y, Ge Z, Duan H, Zou Y, et al Transfer of bone-marrow-derived mesenchymal stem cells influences vascular remodeling and calcification after balloon injury in hyperlipidemic rats. Journal of biomedicine & biotechnology. 2012;2012: 165296. 22665980 10.1155/2012/165296 PMC3361346

[22] Williams AR, Hare JM. Mesenchymal stem cells: biology, pathophysiology, translational findings, and therapeutic implications for cardiac disease. Circulation research. 2011;109: 923–940. 10.1161/CIRCRESAHA.111.243147 21960725 PMC3604746

[23] Ferro F, Spelat R, D'Aurizio F, Falini G, De Pol I, Pandolfi M, et al Acellular bone colonization and aggregate culture conditions diversely influence murine periosteum mesenchymal stem cell differentiation potential in long-term in vitro osteoinductive conditions. Tissue Eng Part A. 2012;18: 1509–1519. 10.1089/ten.TEA.2011.0411 22494486

[24] Pittenger MF, Mackay AM, Beck SC, Jaiswal RK, Douglas R, Mosca JD, et al Multilineage potential of adult human mesenchymal stem cells. Science. 1999;284: 143–147. 10102814 10.1126/science.284.5411.143

[25] Lange C, Schroeder J, Stute N, Lioznov MV, Zander AR. High-potential human mesenchymal stem cells. Stem cells and development. 2005;14: 70–80. 15725746 10.1089/scd.2005.14.70

[26] Li X, Yu X, Lin Q, Deng C, Shan Z, Yang M, et al Bone marrow mesenchymal stem cells differentiate into functional cardiac phenotypes by cardiac microenvironment. Journal of molecular and cellular cardiology. 2007;42: 295–303. 16919679 10.1016/j.yjmcc.2006.07.002

[27] Crisan M. Transition of mesenchymal stem/stromal cells to endothelial cells. Stem cell research & therapy. 2013;4: 95. 23953698 10.1186/scrt306 PMC3854778

[28] Zander AR, Lange C, Westenfelder C. Mesenchymal stromal cells: main factor or helper in regenerative medicine? Kidney Int Suppl. 2011;1: 74–76. 25018905 10.1038/kisup.2011.17 PMC4089635

[29] Uccelli A, Moretta L, Pistoia V. Mesenchymal stem cells in health and disease. Nature reviews Immunology 2008;8: 726–736. 10.1038/nri2395 19172693

[30] Lennon DP, Caplan AI. Isolation of human marrow-derived mesenchymal stem cells. Experimental hematology. 2006;34: 1604–1605. 17046583 10.1016/j.exphem.2006.07.014

[31] Bartholomew A, Sturgeon C, Siatskas M, Ferrer K, McIntosh K, Patil S, et al Mesenchymal stem cells suppress lymphocyte proliferation in vitro and prolong skin graft survival in vivo. Experimental hematology. 2002;30: 42–48. 11823036 10.1016/s0301-472x(01)00769-x

[32] Martin-Rendon E, Brunskill SJ, Hyde CJ, Stanworth SJ, Mathur A, Patil S, et al Autologous bone marrow stem cells to treat acute myocardial infarction: a systematic review. European heart journal. 2008;29: 1807–1818. 10.1093/eurheartj/ehn220 18523058

[33] Gnecchi M, Danieli P, Cervio E. Mesenchymal stem cell therapy for heart disease. Vascular pharmacology. 2012; 57: 48–55. 10.1016/j.vph.2012.04.002 22521741

[34] Fan CQ, Leu S, Sheu JJ, Zhen YY, Tsai TH, Chen YL, et al Prompt Bone Marrow-Derived Mesenchymal Stem Cell Therapy Enables Early Porcine Heart Function Recovery from Acute Myocardial Infarction. International heart journal. 2014;55: 362–371. 24965596 10.1536/ihj.14-007

[35] Rodrigo SF, van Ramshorst J, Hoogslag GE, Boden H, Velders MA, Cannegieter SC, et al Intramyocardial injection of autologous bone marrow-derived ex vivo expanded mesenchymal stem cells in acute myocardial infarction patients is feasible and safe up to 5 years of follow-up. J Cardiovasc Transl Res. 2013;6: 816–825. 10.1007/s12265-013-9507-7 23982478 PMC3790917

[36] Chen S, Liu Z, Tian N, Zhang J, Yei F, Duan B, et al Intracoronary transplantation of autologous bone marrow mesenchymal stem cells for ischemic cardiomyopathy due to isolated chronic occluded left anterior descending artery. J Invasive Cardiol. 2006;18: 552–556. 17090821

[37] Heldman AW, DiFede DL, Fishman JE, Zambrano JP, Trachtenberg BH, Karantalis V, et al Transendocardial mesenchymal stem cells and mononuclear bone marrow cells for ischemic cardiomyopathy: the TAC-HFT randomized trial. JAMA: the journal of the American Medical Association. 2014;311: 62–73. 10.1001/jama.2013.282909 24247587 PMC4111133

